# PDBe PISA: Enhanced Analysis of Macromolecular Interactions

**DOI:** 10.1002/cpz1.70446

**Published:** 2026-09-25

**Authors:** Grisell Díaz Leines, Paulyna Magaña, Sreenath Nair, Jennifer Fleming, Mihaly Varadi, Eugene Krissinel, Sameer Velankar

**Affiliations:** ^1^ Protein Data Bank in Europe, European Molecular Biology Laboratory, European Bioinformatics Institute (EMBL‐ EBI) Wellcome Genome Campus Hinxton Cambridge United Kingdom; ^2^ Scientific Computing Department, Science and Technology Facilities Council Research Complex at Harwell Didcot Oxfordshire United Kingdom

**Keywords:** macromolecular interactions, PDBe API, PISA, protein assemblies, protein interfaces, Protein Data Bank (PDB), structural bioinformatics

## Abstract

Protein interactions play a pivotal role in determining the biological functions of living cells. Gaining structural insights into protein interfaces can illuminate their role in function and diseases and highlight their potential as therapeutic targets. In response, there has been a marked increase in efforts by the scientific community to decipher the nuances of macromolecular interfaces, aiming to predict interactions and binding specificity of different components within complexes. The “Proteins, Interfaces, Structures, and Assemblies” (PISA) software has become a standard tool for analyzing intermolecular interactions and elucidating macromolecular assemblies from molecular structures. This article presents detailed protocols for using Protein Data Bank in Europe (PDBe) PISA API, a new Application Programming Interface (API) for enhanced analysis of protein assembly interfaces, using an updated version of PISA software. Compared to its predecessor, the updated PISA software expands interface data, increasing the coverage and detail of interaction information for assemblies and introduces greater computational flexibility, allowing users to perform targeted interface analyses for selected assemblies efficiently. The PDBe team uses the PISA API to provide interaction data in a JavaScript Object Notation (JSON) format for weekly Protein Data Bank (PDB) releases to support the efforts of the broader scientific community. Our protocols are a comprehensive guide to harnessing macromolecular interaction data using PISA. We delve into practical examples, from querying interaction and interface data from JSON files to extracting interaction data for assemblies cataloged in the PDB archive using programmatic access and bulk download via File Transfer Protocol (FTP). © 2026 The Author(s). *Current Protocols* published by Wiley Periodicals LLC.

**Basic Protocol 1**: Programmatic access to interaction data with PDBe PISA API

**Basic Protocol 2**: FTP access to interaction data of assemblies

**Basic Protocol 3**: Analysis of assembly interfaces and interactions

## INTRODUCTION

Macromolecular complexes play a pivotal role in orchestrating most biological functions within living cells. Intricate biological processes are predominantly driven by protein interactions with other molecular entities. Consequently, an in‐depth analysis of structural features and protein interactions is vital in decoding their biological roles and binding dynamics. Recent advances in experimental methods, such as cryo‐electron microscopy (cryo‐EM), alongside transformative developments in artificial intelligence, most notably the structure prediction AI systems (Abramson et al., 2024; Jumper et al., 2021), have driven an unprecedented expansion in the number of macromolecular structures archived in the Protein Data Bank (PDB) (Berman et al., 2003; wwPDB consortium et al., 2019) and the AlphaFold Protein Structure Database (Fleming et al., 2025; Varadi et al., 2022). Although AlphaFold has revolutionized the accurate prediction of both monomeric and multimeric protein structures, the structural characterization and prediction of macromolecular complexes remains a major challenge, particularly in cases where protein–protein interactions (PPIs) are not known a priori, or dynamic conformational states are involved. These challenges not only reflect the intrinsic complexity of biomolecular interfaces but also highlight the urgent need for high‐quality structural and interaction data to advance both our understanding of macromolecular function and the accurate prediction of protein complexes.

Protein interaction data repositories play a crucial role in supporting structural biology and computational modeling. They enable researchers to identify common structural motifs, assess physicochemical properties of interfaces, detect biologically relevant complexes, and predict PPIs and binding sites (Shin et al., 2020). As the global archive of experimentally determined structures, the PDB provides the foundational data necessary for the classification and prediction of macromolecular assemblies. The Protein Data Bank in Europe (PDBe), a core member of the worldwide PDB (wwPDB) consortium, extends this effort through specialized data services focused on macromolecular complexes (Appasamy et al., 2025; Armstrong et al., 2019; Querino Lima Afonso et al., 2025), including the widely used Proteins, Interfaces, Structures, and Assemblies (PISA) software (Krissinel & Henrick, 2005, 2007). Together with the Complex Portal (Meldal et al., 2015) and other public repositories, such tools represent a comprehensive ecosystem for the prediction, analysis, and visualization of PPIs and complex data, drawing from both experimental data and computational predictions. (Das, & Krishna, 2016; Faure et al., 2012; Hermjakob, 2004; Lawson et al., 2016; Lensink et al., 2025; Mosca et al., 2014; Tuncbag et al., 2008; Valentini et al., 2015; Xue et al., 2015)

A range of computational methods have been developed to identify and analyze macromolecular interfaces from X‐ray crystallography and cryo‐EM structures (Henrick, 1998; Krissinel & Henrick, 2005, 2007; Ponstingl et al., 2003). Among these, PISA (Krissinel, 2010; Krissinel & Henrick, 2005, 2007) is a widely adopted tool for identifying interfaces and inferring relevant assemblies by scoring them based on their calculated thermodynamic stability. The PISA services, hosted by PDBe and CCP4 (Agirre et al., 2023), provide precomputed interface and assembly data for the entire PDB archive in a searchable database while supporting user submissions of custom atomic coordinate files. For crystal structures, PISA expands the crystal symmetry and analyzes interfaces between macromolecules to identify a probable quaternary structure, or it can analyze the interfaces in the uploaded coordinates of a given assembly. The user interface of the PISA servers displays information for each component in a pair of contacting chains, including the total accessible and buried surface area, interfacial area, atoms and residues in the interface, disulfide and hydrogen bonds, salt bridges and covalent bonds in the interface, and interfacial free energy changes, among other information.

To enable scalable and comprehensive analysis of macromolecular interfaces across the expanding PDB archive, we present an updated version of the PISA software, developed in collaboration between the PDBe and CCP4 teams. This enhanced implementation is optimized for large‐scale computation of enriched interaction data for all annotated assemblies in the PDB. This update focuses exclusively on identified assemblies within PDB entries and expands the output to include additional information on individual interfaces, including extended atom‐atom interaction types, while lowering computational overhead. The updated PISA version 2.1.2 is now distributed as part of CCP4 version 9.0 and is used by PDBe to generate enriched macromolecular interaction data on the annotated assemblies in the PDB archive. These data are provided as JavaScript Object Notation (JSON) files via the PDBe public File Transfer Protocol (FTP) area (https://ftp.ebi.ac.uk/pub/databases/msd/pdb‐assemblies‐analysis/) and via the programmatic access endpoints of the PDBe Application Programming Interface (API) (Nair et al., 2021).

The methods described in this article are designed to accommodate varying levels of user expertise. We present comprehensive protocols detailing three complementary approaches for accessing and analyzing macromolecular interaction data:

(1) Programmatic access to the PISA API endpoints (Basic Protocol 1) for on‐demand retrieval of interaction data for specific or small sets of PDB entries
(2) Data retrieval via FTP (Basic Protocol 2) for bulk downloading of interaction data across multiple entries.
(3) Calculation of macromolecular interaction data for a PDB assembly using the updated version of PISA, the latest iteration of the software (Basic Protocol 3), designed for analyzing structures outside the PDB archive, including unpublished experimental data, computational models, or modified coordinates.

We also provide a Python notebook that interactively demonstrates data retrieval from the PDBe API. To ensure reproducibility, the notebook is available as a versioned Git tag (pisa/v1.0.0) in the pdbe‐notebooks repository and can be accessed through Google Colab at: https://github.com/PDBeurope/pdbe‐notebooks/blob/pisa/v1.0.0/pdbe_pisa_APIs/pdbe_PISA_APIs.ipynb.

## Basic Protocol 1 PROGRAMMATIC ACCESS TO INTERACTION DATA WITH PDBe PISA API

Programmatic access to PISA data via the PDBe API enables scalable, reproducible analysis of macromolecular assemblies and interfaces across the PDB archive. The API provides structured JSON outputs that can be directly integrated into automated workflows, large‐scale surveys, and downstream computational analyses. This enables users to systematically assess assembly stability, compare interfaces within and across structures, and aggregate interaction features such as buried surface area, thermodynamic parameters, and atomic‐level contacts.

The weekly synchronization of the API with the PDB release cycle ensures that analyses remain consistent with the current archive, supporting reproducibility and longitudinal analyses. This protocol describes how to use PDBe API endpoints to obtain assembly and interaction data for a given PDB entry; for a specific assembly, a single interface, and all assembly interfaces.

We provide sample codes to retrieve assembly and interface data for the Yeast 20S Proteasome (PDB: 1iru), a large 28‐subunit complex with D7 symmetry. To know more about this PDB entry, go to the summary page at: https://www.ebi.ac.uk/pdbe/entry/pdb/1iru.

The parameters and data structures of the endpoints are available from Apiary at: https://pdbeapi.docs.apiary.io/.

### Necessary Resources

#### Hardware

A computer with a stable internet connection and free drive space to store data

##### Software

Python 3.10+ (https://www.python.org/downloads/)

Python ‘requests’ library (https://requests.readthedocs.io/en/latest/user/install/)

##### Files

None

1 Get data for a single assembly.
  a. Users can retrieve data for a single assembly. The specified API endpoints return JSON‐formatted responses for a requested assembly based on the provided PDB entry (:*pdbid*) and assembly ID (:*assemblyid*) and must follow the syntax www.ebi.ac.uk/pdbe/api/pisa/assembly/:pdbid/:assemblyid.
  The following code will send a request to get assembly details for entry *pdbid* = 1iru and the preferred assembly, *assemblyid* = 1.
  Sample code:


import requests, sys, json
import pandas as pd
API_point = 'https://www.ebi.ac.uk/pdbe/api/pisa/assembly/
'query_pdbid = '1iru'
query_assembly_id = '1'
response = requests.get(f'{API_point}{query_pdbid}/{query_assembly_id}')
if response.status_code == 200:
print(json.dumps(response.json(), indent=3))

  Output:


{
"1iru": {
"assembly_id": "1",
"pisa_version": "2.0",
"assembly": {
"id": "1",
"size": "56",
"interface_count": 82,
"score": "",
"macromolecular_size": "28",
"dissociation_energy": 69.0,
"accessible_surface_area": 212622.4,
"buried_surface_area": 113317.18,
"entropy": 28.55,
"dissociation_area": 7116.05,
"solvation_energy_gain": ‐637.29,
"number_of_uc": "0",
"number_of_dissociated_elements": "3",
"symmetry_number": "2",
"formula": "\n A(2)B(2)C(2)D(2)E(2)F(2)G(2)H(2)I(2)J(2)K(2)L(2)M(2)N(2)a(28)\n ",
"composition": "AOBPCQDRESFTGUHVIWJXKYLZ1M2N[MG](28)",
"R350": ""
}
}
}

  The JSON response includes parameters detailing structural and thermodynamic properties of the assembly. Specific information can be extracted from this JSON response, as shown in the following code.
  Sample code:


response_data = response.json()
pdb_id = list(response_data.keys())[0]
assembly_data = response_data[pdb_id]['assembly']
total_size = assembly_data.get('size', 'N/A')
macromolecular_size = assembly_data.get('macromolecular_size', 'N/A')
buried_surface_area = assembly_data.get('buried_surface_area', 'N/A')
accessible_surface_area = assembly_data.get('accessible_surface_area', 'N/A')
no_interfaces = assembly_data.get('interface_count', 'N/A')
dissociation_energy = assembly_data.get('dissociation_energy', 'N/A')
entropy = assembly_data.get('entropy', 'N/A')
print(f"--- Assembly Summary for {pdb_id} (Assembly {query_assembly_id}) ---")
print(f" Components: {total_size} total ({macromolecular_size}‐mer protein chains)")
print(f" Interfaces: {no_interfaces} total")
print(f" Accessible Surface Area: {accessible_surface_area} Å²")
print(f" Buried Surface Area: {buried_surface_area} Å²")
print(f" Dissociation Energy (ΔGdiss): {dissociation_energy} kcal/mol")
print(f" Entropy: {entropy} kcal/mol")
if not response_data:
print("Cannot parse data, the request was not successful or returned empty data.")

  Output:


--- Assembly Summary for 1iru (Assembly 1) ---
Components: 56 total (28‐mer protein chains)
Interfaces: 82 total
Accessible Surface Area: 212622.4 Å²
Buried Surface Area: 113317.18 Å²
Dissociation Energy (ΔGdiss): 69.0 kcal/mol
Entropy: 28.55 kcal/mol

  The JSON output provides the primary metrics for assessing the overall assembly. For 1iru, the output confirms a 28‐mer protein complex with 82 total interfaces. The calculated dissociation energy indicates a stable obligate complex, aligning perfectly with the known biology of the 20S proteasome as a robust molecular machine. For detailed parameter definitions, see the Understanding Results section below).
  b. While we focus on a single entry, the programmatic nature of the API allows for querying multiple similar assemblies, such as 1ryp, 1iru, and 5le5, to compare properties.
  Sample code:


query_pdbids = ['1iru', '1ryp', '5le5']
query_assembly_id = '1'
assembly_results = []
for pdb_id in query_pdbids:
response = requests.get(f'{API_point}{pdb_id}/{query_assembly_id}')
if response.status_code == 200:
response_data = response.json()
assembly_data = response_data[pdb_id]['assembly']
assembly_results.append({
'PDB ID': pdb_id,
'Assembly ID': query_assembly_id,
'Macromolecular Size': f"{assembly_data.get('macromolecular_size', 'N/A')}‐mer",
'Interfaces': assembly_data.get('interface_count', 'N/A'),
'Buried Surface Area (Å²)': assembly_data.get('buried_surface_area', 'N/A'),
'ΔGdiss (kcal/mol)': assembly_data.get('dissociation_energy', 'N/A')
})
df_assemblies = pd.DataFrame(assembly_results)
print(df_assemblies.to_string(index=False))

  Output:

**Table:** 

PDB ID	Assembly ID	Macromolecular Size	Interfaces	Buried Surface Area (Å²)	ΔGdiss (kcal/mol)
1iru	1	28‐mer	82	113317.18	69.00
1ryp	1	28‐mer	92	120757.64	50.28
5le5	1	28‐mer	84	127121.21	44.56
2 Retrieving single interface data in an assembly.
  a. The second PDBe API endpoint for macromolecular interfaces provides a JSON response for a single interface selected within an assembly based on a given PDB assembly and an interface ID. Descriptions of the interface attributes are available at: https://pdbeapi.docs.apiary.io.
  The API endpoint pattern is: www.ebi.ac.uk/pdbe/api/pisa/interface/:pdbid/:assemblyid/:interfaceid.
  For instance, the assembly in PDB entry 1iru with assemblyid 1 has 82 interfaces. Users can access details of each interface with the interface ID, which is a 1‐indexed ordinal number, e.g., interfaceid 25.
  Sample code:


import requests, sys, json
API_point_single_interface = "https://www.ebi.ac.uk/pdbe/api/pisa/interface/"
query_pdbid = '1iru'
query_assemblyid = '1'
query_interfaceid = '25'
response_single_interface = requests.get(f'{API_point_single_interface}{query_pdbid}/{query_assemblyid}/{query_interfaceid}')
if response_single_interface.status_code == 200:
interface_data = response_single_interface.json()
print(f"— Interface {query_interfaceid} Summary ---")
print(f" Interface area: {interface_data.get('interface_area')} Å²")
print(f" Solvation energy (ΔGsolv):: {interface_data.get('solvation_energy')} kcal/mol")
print(f" Stabilization energy (ΔGstab):: {interface_data.get('stabilization_energy')} kcal/mol")
print(f" Hydrogen bonds: {interface_data.get('number_hydrogen_bonds')}")
print(f" Salt bridges: {interface_data.get('number_salt_bridges')}")
print(f" p‐value: {interface_data.get('p_value')}")
molecules = interface_data.get('molecules', [])
chains = [molecule.get('chain_id') for molecule in molecules]
print(f" Interacting chains: {chains}")

  Output:


--- Interface 25 Summary ---
Interface area: 766.45 Å²
Solvation energy (ΔGsolv): ‐2.15 kcal/mol
Stabilization energy (ΔGstab): ‐9.85 kcal/mol
Hydrogen bonds: 14
Salt bridges: 4
p‐value: 0.572
Interacting chains: ['X', 'L']

  This endpoint provides granular details on a specific pairwise interaction. The *p*‐value is a measure of interface specificity, representing the probability of observing a solvation energy gain (ΔGs) lower than for randomly picked atoms from the protein surface. For interface 25, the *p*‐value of 0.572 (where *p* > .5) indicates that the interface is less hydrophobic than expected by chance (ΔGs > ΔGavg). This suggests the interaction is likely an artefact of crystal packing rather than a biologically specific contact. Such results warrant further visual inspection of the crystal lattice to distinguish true biological interactions from packing effects.
  b. With all the interface details in this JSON response, users can extract information about the number of interface residues or specific contacts, such as hydrogen bonds.
  Sample code:


import requests, sys, json
import pandas as pd
API_point_single_interface = "https://www.ebi.ac.uk/pdbe/api/pisa/interface/"
query_pdbid = '1iru'
query_assemblyid = '1'
query_interfaceid = '25'
response_single_interface = requests.get(f'{API_point_single_interface}{query_pdbid}/{query_assemblyid}/{query_interfaceid}')
interface_data = response_single_interface.json()
interface_id = interface_data.get('interface_id')
no_interface_residues = interface_data.get('number_interface_residues')
no_hydrogen_bonds = interface_data.get('number_hydrogen_bonds')
no_salt_bridges = interface_data.get('number_salt_bridges')
print(f"The interface {interface_id} of assembly {query_pdbid} has {no_interface_residues} interface residues with {no_hydrogen_bonds} hydrogen bonds and {no_salt_bridges} salt bridges.")

  Output:


The interface 25 of assembly 1iru has 201 interface residues with 14 hydrogen bonds and 4 salt bridges.

  c. We can then extract information for the specific type of contacts.
  Sample code:


import requests, sys, json
import pandas as pd
from IPython.display import display
API_point_single_interface = "https://www.ebi.ac.uk/pdbe/api/pisa/interface/"
query_pdbid = '1iru'
query_assemblyid = '1'
query_interfaceid = '25'
response_single_interface = requests.get(f'{API_point_single_interface}{query_pdbid}/{query_assemblyid}/{query_interfaceid}')
interface_data = response_single_interface.json()
if response_single_interface.status_code == 200:
interface_pair_prop = []
if "hydrogen_bonds" in interface_data:
prop = interface_data["hydrogen_bonds"]
label_seq_atom_1 = prop.get("atom_site_1_label_seq_ids", [])
label_id_atom_1 = prop.get("atom_site_1_label_atom_ids", [])
chain_atom_1 = prop.get("atom_site_1_chains", [])
bond_distances = prop.get("bond_distances", [])
label_seq_atom_2 = prop.get("atom_site_2_label_seq_ids", [])
label_id_atom_2 = prop.get("atom_site_2_label_atom_ids", [])
chain_atom_2 = prop.get("atom_site_2_chains", [])
for (c1, r1, a1, c2, r2, a2, dist) in zip(
chain_atom_1, label_seq_atom_1, label_id_atom_1,
chain_atom_2, label_seq_atom_2, label_id_atom_2,
bond_distances # Zip in the distances
):
interface_pair_prop.append([f"{c1}:{r1}", a1.strip(),
dist,
f"{c2}:{r2}", a2.strip()])
df_hbonds = pd.DataFrame(
interface_pair_prop, columns=[
"Chain:ResID (Atom 1)", "Atom (Atom 1)",
"Distance (Å)",
"Chain:ResID (Atom 2)", "Atom (Atom 2)"])
print(f"\nHydrogen Bonds for Interface {query_interfaceid}:")
display(df_hbonds)
else:
print("Cannot extract H‐bonds, the request was not successful.")

  Output:
  The code output is a formatted table displaying the extracted hydrogen bonds for interface 25.
  d. The API follows a consistent nested structure for all contact types. To retrieve Salt Bridges, you can reuse the logic above by updating the data source. Simply change the key from “hydrogen_bonds” to “salt_bridges”:

if "salt_bridges" in interface_data:
sb_prop = interface_data["salt_bridges"]

  e. You can retrieve a filtered list of interface residues for a specific interface (interface 25), focusing on residues with buried surface area, energetic contribution, or explicit intermolecular interactions.
    Note that the number of residues reported by PISA for an interface includes all residues assigned to the interface, whereas the table shown here includes only residues meeting the filtering criteria described above.
  Sample code:


import requests
import pandas as pd
from IPython.display import display
API_point_single_interface = "https://www.ebi.ac.uk/pdbe/api/pisa/interface/" query_pdbid = '1iru'
query_assemblyid = '1'
query_interfaceid = '25'
response_single_interface = requests.get(f'{API_point_single_interface}{query_pdbid}/{query_assemblyid}/{query_interfaceid}')
if response_single_interface.status_code == 200
interface_data = response_single_interface.json()
interface_id = interface_data.get('interface_id')
no_interface_residues = interface_data.get('number_interface_residues')
no_hydrogen_bonds = interface_data.get('number_hydrogen_bonds')
no_salt_bridges = interface_data.get('number_salt_bridges')
print(f"The interface {interface_id} of assembly {query_pdbid} has {no_interface_residues} interface residues with {no_hydrogen_bonds} hydrogen bonds and {no_salt_bridges} salt bridges.\n")
interface_residues_data = []
if 'molecules' in interface_data:
for molecule in interface_data['molecules']:
chain_id = molecule.get('chain_id', '')
molecule_class = molecule.get('molecule_class', '')
residues = molecule.get('residue_label_comp_ids', [])
seq_ids = molecule.get('residue_label_seq_ids', [])
asa_values = molecule.get('accessible_surface_areas', [])
bsa_values = molecule.get('buried_surface_areas', [])
solvation_energies = molecule.get('solvation_energies', [])
residue_bonds = molecule.get('residue_bonds', [])
for i, (residue, seq_id) in enumerate(zip(residues, seq_ids)):
# Only include residues that are part of the interface (BSA > 0 or involved in bonds)
asa = asa_values[i] if i < len(asa_values) else 0
bsa = bsa_values[i] if i < len(bsa_values) else 0
dg = solvation_energies[i] if i < len(solvation_energies) else 0
bond_type = residue_bonds[i] if i < len(residue_bonds) else ''
if bsa > 0 or bond_type or abs(dg) > 0.01:
interface_residues_data.append({
'Structure': f"{chain_id}:{residue}:{seq_id}",
'Chain': chain_id,
'Residue': residue,
'ResID': seq_id,
'H': 'H' if 'H' in str(bond_type) else '',
'S': 'S' if 'S' in str(bond_type) else '',
'D': 'D' if 'D' in str(bond_type) else '',
'C': 'C' if 'C' in str(bond_type) else '',
'ASA': round(asa, 2),
'BSA': round(bsa, 2),
'ΔGi': round(dg, 2)
})
df_interface_residues = pd.DataFrame(interface_residues_data)
if not df_interface_residues.empty:
df_interface_residues['ResID_num'] = pd.to_numeric(df_interface_residues['ResID'], errors='coerce')
df_interface_residues = df_interface_residues.sort_values(['Chain', 'ResID_num']).drop('ResID_num', axis=1)
df_interface_residues.reset_index(drop=True, inplace=True)
df_interface_residues.index += 1
print(f"All Interface Residues for Interface {query_interfaceid}:")
display(df_interface_residues[['Structure', 'H', 'S', 'D', 'C', 'ASA', 'BSA', 'ΔGi']])
else:
print("No interface residues found in the response.")
else:
print(f"Request failed with status code: {response_single_interface.status_code}")

  Output:
  The output presents a table that lists interface residues with non‐zero buried surface area, measurable solvation energy contribution, or involvement in specific intermolecular interactions.
3 Retrieve all interfaces in an assembly.
  The third API endpoint returns a JSON response containing details of all the interfaces within an assembly based on a given PDB entry and assembly ID.
  The data encompasses structural and calculated thermodynamic parameters for the assembly and interface. The API endpoint pattern for retrieving this data is: www.ebi.ac.uk/pdbe/api/pisa/interfaces/:pdbid/:assemblyid/:interfaceid. For example, there are three identified interfaces for PDB entry *1iru* 1 and preferred assembly (*assemblyid*) 1.
  Sample code:


import requests, sys, json
API_point_all_interfaces ="https://www.ebi.ac.uk/pdbe/api/pisa/interfaces/"
query_pdbid = '1iru'
query_assemblyid = '1'
response_all_interfaces = requests.get(f'{API_point_all_interfaces}{query_pdbid}/{query_assemblyid}')
all_data = response_all_interfaces.json()
num_interfaces = all_data[query_pdbid]['assembly']['interface_count']
print(f"There are {num_interfaces} interfaces")

  Output:


There are 82 interfaces

  Alternatively, the complete JSON can be retrieved with:


print(json.dumps(response_all_interfaces.json(), indent=5))

  With comprehensive interface details available for all interfaces in an assembly, the data can be aggregated into a comparative table to support systematic analysis. Rather than inspecting interfaces individually, this representation enables users to evaluate all interfaces in the context of the complete assembly.
  Sample code:


import pandas as pd
from tabulate import tabulate
import requests, sys, json
from IPython.display import display
def analyze_interface(interface_data):
analysis = {}
analysis['interface_id'] = interface_data.get('interface_id', None)
analysis['interface_area'] = interface_data.get('interface_area', 0.0)
analysis['stabilization_energy'] = interface_data.get('stabilization_energy', 0.0)
analysis['solvation_energy'] = interface_data.get('solvation_energy', 0.0)
analysis['number_hydrogen_bonds'] = interface_data.get('number_hydrogen_bonds', 0)
analysis['number_salt_bridges'] = interface_data.get('number_salt_bridges', 0)
analysis['p_value'] = interface_data.get('p_value', None)
molecules = interface_data.get('molecules', [])
mol_classes = set(
molecule.get('molecule_class', 'Unknown')
for molecule in molecules
)
sorted_classes = sorted(list(mol_classes))
if len(sorted_classes) == 1:
analysis['interaction_type'] = f"{sorted_classes[0]}‐{sorted_classes[0]}"
else:
analysis['interaction_type'] = '-'.join(sorted_classes)
chains = sorted(list(set(
molecule.get('chain_id')
for molecule in molecules
if molecule.get('chain_id') is not None
)))
analysis['interacting_chains'] = ', '.join(chains)
return analysis
def compare_interfaces(assembly_data):
interfaces = assembly_data.get('interfaces', [])
comparison_data = []
for interface in interfaces:
analysis = analyze_interface(interface)
analysis['interface_id'] = interface.get('interface_id')
comparison_data.append(analysis)
df = pd.DataFrame(comparison_data)
df = df.set_index('interface_id')
return df
if response_all_interfaces.status_code == 200:
assembly_full_data = all_data[query_pdbid]['assembly']
df_comparison = compare_interfaces(assembly_full_data)
print(f"\n{len(df_comparison)} Interfaces in {query_pdbid}")
display(df_comparison)
else:
print("Cannot run comparison, the all‐interfaces request was not successful.")

  Output (first 10 rows):
  This comparative table is crucial for distinguishing biologically significant interfaces from weaker crystal contacts. Interface 1 (Chains I, J) has a large interface area (1473.4 Å²), a highly favorable stabilization energy (–31.99 kcal/mol), and a *p*‐value = .151, strongly indicating a specific, stable interaction.

**Table:** 

**interface_id**	**interface_area**	**stabilization_energy**	**solvation_energy**	**number_hydrogen_bonds**	**number_salt_bridges**	**p_value**	**interaction_type**	**interacting_chains**
**1**	1473.4	−31.99	−19.39	20	10	0.151	Protein‐Protein	I, J
**2**	1472.72	−32.1	−19.5	20	10	0.158	Protein‐Protein	W, X
**3**	1368.82	−17.23	−5.67	21	6	0.741	Protein‐Protein	Q, R
**4**	1363.18	−17.12	−5.57	21	6	0.749	Protein‐Protein	C, D
**5**	1352.64	−21.81	−12.92	15	6	0.378	Protein‐Protein	O, P
**6**	1349.26	−21.28	−12.76	15	5	0.386	Protein‐Protein	A, B
**7**	1345.75	−22.56	−14.41	15	4	0.262	Protein‐Protein	P, Q
**8**	1343.77	−22.68	−14.46	16	3	0.257	Protein‐Protein	B, C
**9**	1336.0	−21.52	−10.05	25	1	0.347	Protein‐Protein	F, G
**10**	1317.6	−20.07	−9.93	22	1	0.321	Protein‐Protein	T, U
  To identify interaction hotspots, we perform a residue‐level aggregation of specific intermolecular contacts across all detected interfaces in the assembly. For each interface, residues participating in hydrogen bonds and salt bridges are extracted from the PISA interaction annotations and represented by their chain identifier and UniProt residue number.
  Residues are then counted based on their frequency of occurrence in these interactions across interfaces. In this context, interaction “hotspots” are defined as residues that repeatedly participate in hydrogen bonding or salt bridge formation, indicating persistent involvement in stabilizing intermolecular contacts within the assembly.
  This frequency‐based analysis does not quantify interaction strength or energetic contribution but provides a simple and transparent way to prioritize residues that may play a structurally important role at the interface and merit further structural or functional investigation.
  Sample code:


assembly_data = all_data[query_pdbid]['assembly']
interfaces = assembly_data.get('interfaces',[])
# Initialise lists to store all interacting residues
from collections import Counter
all_hbond_residues = []
all_salt_bridge_residues = []
# Loop through each interface
for interface in interfaces:
interface_id = interface.get('interface_id')
# Get residues from HYDROGEN BONDS
if "hydrogen_bonds" in interface:
prop = interface["hydrogen_bonds"]
# Get chain and UNP number for a more unique ID
chains_1 = prop["atom_site_1_chains"]
unp_nums_1 = prop["atom_site_1_unp_nums"]
chains_2 = prop["atom_site_2_chains"]
unp_nums_2 = prop["atom_site_2_unp_nums"]
# Add as tuples (Chain, ResNum)
all_hbond_residues.extend([(f"Chain {c}", n) for c, n in zip(chains_1, unp_nums_1)])
all_hbond_residues.extend([(f"Chain {c}", n) for c, n in zip(chains_2, unp_nums_2)])
# Get residues from SALT BRIDGES
if "salt_bridges" in interface:
prop = interface["salt_bridges"]
chains_1 = prop["atom_site_1_chains"]
unp_nums_1 = prop["atom_site_1_unp_nums"]
chains_2 = prop["atom_site_2_chains"]
unp_nums_2 = prop["atom_site_2_unp_nums"]
all_salt_bridge_residues.extend([(f"Chain {c}", n) for c, n in zip(chains_1, unp_nums_1)])
all_salt_bridge_residues.extend([(f"Chain {c}", n) for c, n in zip(chains_2, unp_nums_2)])
# Count the frequency of each residue
hbond_counts = Counter(all_hbond_residues)
salt_bridge_counts = Counter(all_salt_bridge_residues)
# Convert to DataFrames for nice printing
df_hbond_hotspots = pd.DataFrame(hbond_counts.most_common(), columns=["Residue (Chain, UNP Num)", "H‐Bond Count"])
df_salt_bridge_hotspots = pd.DataFrame(salt_bridge_counts.most_common(), columns=["Residue (Chain, UNP Num)", "Salt Bridge Count"])
print("--- Hydrogen Bond Hotspots ---)
display(df_hbond_hotspots)
print("--- Salt Bridge Hotspots---")
display(df_salt_bridge_hotspots)

  Output:
  The output consists of tables listing residues (identified by chain and UniProt residue number) ranked by the number of hydrogen bonds or salt bridges in which they participate across all interfaces. The counts indicate how often each residue is observed with a specific interaction type. While they may indicate structural importance, further analysis is required to distinguish symmetry‐driven effects from unique functional roles.

## Basic Protocol 2 FTP ACCESS TO INTERACTION DATA OF ASSEMBLIES

This protocol can be followed for bulk downloading all pre‐calculated interaction data from the FTP area, a recommended method if many entries need to be downloaded (e.g., more than 10 to 20) or to obtain the entire database.

All the pre‐calculated interaction data generated with PISA for all the assemblies in the PDB archive are available in the public FTP area of EMBL‐EBI at: https://ftp.ebi.ac.uk/pub/databases/msd/pdb‐assemblies‐analysis/split/. This area is updated weekly with the public release of the PDB archive. Interface parameters, encompassing elements such as interface contacts, hydrogen and disulfide bonds, salt bridges, covalent bonds, van der Waals contacts, and assembly parameters (e.g., accessible and buried surface areas, dissociation energies, and contact interface characteristics) are provided in JSON format.

The availability of all assembly and interface data in a consistent JSON format facilitates automated, high‐throughput analysis. PDB entries are grouped in folders named after the second and third characters of the PDB ID. There are two JSON files for each assembly's PDB ID with its corresponding assembly code:

Here we provide details on how to access data for the complete *Escherichia coli* DNA gyrase complex bound to a 130 bp DNA duplex (PDB: 6rkw) via the FTP area.

### Necessary Resources

#### Hardware

A computer with a stable internet connection and free drive space to store data

##### Software

An internet browser

##### Files

None

1 Navigate to the FTP area at: https://ftp.ebi.ac.uk/pub/databases/msd/pdb‐assemblies‐analysis/split/.
2 To access data for the entry “6rkw”, navigate to the folder “rk”, as folders are named based on the second and third characters of the corresponding PDB IDs.
3 Assembly data can be found inside this folder as:


6rkw_assembly1.json
6rkw_assembly1_interfaces.json

  The first file contains the assembly details, and the second file contains all the interfaces and interactions data.

## Basic Protocol 3 ANALYSIS OF ASSEMBLY INTERFACES AND INTERACTIONS

To compute comprehensive interaction data for assemblies in the PDB, we have developed a Python package, pisa‐analysis v3.3.2, which interfaces with the updated PISA v2.1.2 implementation (https://github.com/PDBe‐KB/pisa). This package enables users to run PISA calculations locally, providing code to perform standalone analyses of macromolecular interfaces and interactions using their own coordinate files. This protocol is designed for analyzing macromolecular structures not included in the PDB, such as newly generated models, modified coordinates, or structures awaiting deposition.

The module pisa‐analysis will perform the following tasks:

(1) Analyzes macromolecular interfaces of a given assembly with PISA.
(2) Creates a JSON dictionary with thermodynamic and structural information of an assembly.
(3) Creates a JSON dictionary with enhanced interactions/interfaces data for a given assembly.

This enables researchers to apply the same rigorous and enhanced PISA analysis to their own computational or unpublished experimental data.

### Necessary Resources

#### Hardware

A computer with a stable internet connection, free drive space to store data and the capability of running GEMMI (https://gemmi.readthedocs.io/) and Python 3

##### Software

Python 3.10 (https://www.python.org/downloads/)

Python modules: xmltodict (https://pypi.org/project/xmltodict/) gemmi (https://gemmi.readthedocs.io/en/latest/install.html#python‐module) xmlschema (https://pypi.org/project/xmlschema/) jsonschema (https://pypi.org/project/jsonschema/) lxml (https://pypi.org/project/lxml/) pandas (https://pypi.org/project/pandas/)

If using Linux or Mac OS, you can install all these dependencies using pip (e.g., pip install xmltodict); if using Windows, we recommend downloading and using Git Bash (https://gitforwindows.org/).

##### Files

Assembly CIF file (mmCIF); alternatively PDB‐format file can be used

#### Dependencies

This protocol uses the updated PISA v2.1.2 software, a standalone implementation to compute assemblies and generate enriched information on macromolecular interfaces, mirroring the expanded data produced at PDBe. Users who wish to explore the full capabilities of PISA, including documentation on assembly prediction and interface annotation, are referred to the resources provided in the PISA repository.

Importantly, the workflow described here using the pisa‐analysis v3.3.2 module performs calculations only on specified assemblies (e.g., annotated biological assemblies or user‐specified models). It does not perform assembly prediction.

The pisa‐analysis module runs PISA as a subprocess and therefore requires that PISA v2.1.2 be compiled in advance. Please download and compile PISA v2.1.2 from the repository at: https://github.com/PDBe‐KB/pisa. The necessary compilation steps are outlined below.

1 Clone PISA repository.
  In Linux and MacOS, use the built‐in terminal application. The following set of commands will create a folder containing pisa and pisa‐analysis and clone the pisa repository from GitHub. The third command changes the current working directory to the pisa directory in preparation for further actions within that directory. First, navigate to the directory where PISA was compiled.


mkdir PISA
cd PISA
git clone http://github.com/PDBe‐KB/pisa
cd pisa

2 Compile PISA from source.
  a. Edit the compile.sh file. Navigate to the root directory where pisa was cloned (…/pisa/compile.sh), open the compile.sh, locate the variable ‘srcdir=$SRCDIR’ and update it to the complete path to the pisa directory.

export SRCDIR=/path/to/your/pisa

   
  b. Compile PISA. The code snippet compiles PISA. This line will make the compile.sh script executable and then execute the script. After compilation, the pisa binary will be generated in /path/to/your/pisa/build:

chmod +x compile.sh;./compile.sh

3 Run PISA using Docker.
  As an alternative to compiling PISA from source, users can run PISA v2.1.2 using the official Docker image. This provides a standardized execution environment and improves reproducibility across platforms.
  a. First, install and start Docker by following the instructions for your operating system: https://docs.docker.com/get‐docker/.
  b. From the root directory of the PISA repository, pull the Docker image:

cd /path/to/pisa
docker pull pdbegroup/pisa

   
  c. Run the example analysis:

docker run --rm ‐v "$PWD:/data" pdbegroup/pisa \
pisa test‐session ‐analyse /data/example_data/6gve.cif \
/usr/share/pisa/setup/pisa_cfg_tmp

   
  d. Retrieve the interface information as XML:

docker run --rm ‐v "$PWD:/data" pdbegroup/pisa \
pisa test‐session ‐xml interface /usr/share/pisa/setup/pisa_cfg_tmp >
interfaces.xml

  The analysis creates a test‐session/ directory in the current working directory, and the interface and assembly information are written to interfaces.xml and assembly.xml. If a different session name is used instead of test‐session, the same session name must also be specified in the command used to generate the XML output.
  To analyze your own structure, replace /data/example_data/6gve.cif with the path to your CIF file inside the container. The current working directory is mounted as /data, so a file named my_structure.cif located in the current directory should be referenced as /data/my_structure.cif.
4 Clone the pisa‐analysis repository.
  The following code snippet will navigate back to the parent folder PISA and download the source code of the pisa‐analysis module from the GitHub repository: https://github.com/PDBe‐KB/pisa‐analysis, navigate into the downloaded directory, and then install the module using the provided installation script.


cd PISA
git clone https://github.com/PDBe‐KB/pisa‐analysis
cd pisa‐analysis
python --version
python ‐m venv .venv
source .venv/bin/activate
export PATH="$PATH:/path/to/your/pisa/build"
export PISA_SETUP_DIR="/path/to/your/pisa/setup"
python ‐m pip install ‐r requirements.txt
pisa_analysis –help

5 Run pisa‐analysis.
  a. To run the module on the command line:

pisa_analysis ‐i INPUT_CIF_FILE --pdb_id PDB_ID --assembly_id ASSEMBLY_CODE ‐o OUTPUT_DIR_JSON --output_xml OUTPUT_DIR_XML

  The current repo contains examples; to run them, you need to replace the arguments with:


pisa_analysis ‐i example_data/6nxr‐assembly1.cif.gz --pdb_id 6nxr --assembly_id 1 ‐o example_data --output_xml example_data

  Required arguments:

**Table:** 

--input_cif (‐i)	: Assembly CIF file (It can also read a PDB file)
--pdb_id	: Entry ID
--assembly_id	: Assembly code
--output_json (‐o)	: Output directory for JSON file
--output_xml	: Output directory for XML files
[‐h] help
  Other optional arguments:

**Table:** 

--input_updated_cif	: Updated cif for pdbid entry
--force	: Always runs PISA calculation
--pisa_setup_dir	: Path to the ‘setup’ directory in PISA
--pisa_binary	: Binary file for PISA
  b. Output files: pisa_analysis runs PISA in a subprocess and generates two XML files, which are saved in the output directory defined by the --output_xml argument:

interfaces.xml
assembly.xml

  If the XML files exist and are valid, the process will skip running PISA unless the --force flag is used in the arguments.
  Next, the process parses these XML files and creates a dictionary that contains all assembly interfaces and interactions information. While creating the interface dictionary for the entry, the process reads UniProt accession and sequence IDs from an updated mmCIF file using Gemmi. The process then parses the XML file assembly.xml generated by PISA and creates a simplified dictionary with some assembly information. In the final steps, the process exports the dictionaries into JSON files. The JSON files are saved in the output directory defined by the ‐o or --output_json arguments. The output JSON files are the following, where xxx is the PDB entry ID, and X is the assembly code:


xxx‐assemX‐interfaces.json xxx‐assemblyX.json
  c. Results. The simplified assembly JSON output looks as follows:

{
"6nxr": {
"assembly_id": "1",
"pisa_version": "2.0",
"assembly": {
"id": "1",
"size": "3",
"interface_count": 4,
"score": "",
"macromolecular_size": "1",
"dissociation_energy": ‐1.08,
"accessible_surface_area": 11124.95,
"buried_surface_area": 330.41,
"entropy": 0.83,
"dissociation_area": 101.3,
"solvation_energy_gain": ‐7.83,
"number_of_uc": "0",
"number_of_dissociated_elements": "2",
"symmetry_number": "1",
"formula": "Aab",
"composition": "A[NA][GOL]",
"R350": ""
}
}
}

  The xxx‐assemX‐interfaces.json file will contain detailed information on all interfaces, similar to the JSON output from Basic Protocol 1, step 3.
  For more information on the assembly interfaces JSON file and its schema, please refer to the detailed documentation available at: https://pdbeapi.docs.apiary.io/.
6 Run pisa‐analysis using Docker.
  As an alternative to installing and configuring PISA and pisa‐analysis locally, users can run the complete workflow using the pisa‐analysis Docker image. This approach provides a preconfigured and reproducible environment containing all required software dependencies.
  In this example, we use the input file 6nxr‐assembly1.cif.gz, which is provided in the example_data directory of the pisa‐analysis repository.
  a. Navigate to the directory containing the input file and create directories for the output files:

cd /path/to/your/workdir
mkdir ‐p output_json_dir output_xml_dir

   
  b. Download the latest pisa‐analysis Docker image:

docker pull pdbegroup/pisa‐analysis

   
  c. In the command below, the current working directory is mounted inside the container as /data_dir. Place the input mmCIF file in this directory and run the analysis:

docker run --rm \
‐v "$PWD:/data" \
‐v "$PWD:/data_dir" \
pdbegroup/pisa‐analysis \
pisa_analysis \
--input_cif /data_dir/6nxr‐assembly1.cif.gz \
--pdb_id 6nxr \
–assembly_id 1 \
--output_json /data_dir/output_json_dir \
--output_xml /data_dir/output_xml_dir

   
  d. Replace the following values as appropriate:
    i. 6nxr‐assembly1.cif.gz with the name of the input mmCIF file.
    ii. 6nxr with the identifier used for the structure.
    iii. 1 with the assembly identifier to be analyzed.
    iv. output_json_dir with the directory where JSON output files should be written.
    v. output_xml_dir with the directory where XML output files should be written.
  The XML files contain the raw PISA assembly and interface annotations, whereas the JSON files contain the processed thermodynamic, structural and interaction information generated by pisa‐analysis.

## COMMENTARY

### Background Information

The Proteins, Interfaces, Structures, and Assemblies (PISA) software is a standard tool for identifying intermolecular interfaces and predicting biologically relevant macromolecular assemblies from 3D structural data (Krissinel & Henrick, 2007). PISA evaluates all interfaces within a crystal lattice and reports a range of physicochemical properties, including interface area, solvation energy gain, hydrogen bonds, salt bridges, and the free energy of dissociation [ΔG₍diss₎]. These thermodynamic parameters support the identification and prediction of stable quaternary structures and enable statistical assessment of interface specificity through the complexation significance score (CSS) and its associated p‐value (Krissinel, 2010).

In this article, we use the updated PISA implementation to perform large‐scale computation of macromolecular interaction data at PDBe. This version provides expanded interface annotations and a broadened catalog of atom–atom interaction types, enabling more detailed analyses than the original software release. Distributed as part of CCP4 version 9.0, the implementation available at: https://github.com/PDBe‐KB/pisa, is the same version used by PDBe to generate enriched interaction data for all annotated assemblies in the PDB archive. Full documentation and source code for this updated PISA implementation are available through the CCP4/PDBe repository.

It is important to emphasize that PISA results are computational predictions, and any conclusions must be validated in the context of other biological and experimental analyses. The results from PISA may be used to generate hypotheses and support the validation of independent biochemical data.

This article provides three distinct methods for harnessing PISA data:

(1) Programmatic access to weekly‐updated, pre‐calculated assembly and interface data for all PDB entries via the PDBe PISA API (Basic Protocol 1).
(2) Bulk download of the same data via FTP (Basic Protocol 2).
(3) A standalone PISA package that expands the output data to include enriched information on individual interfaces and extended atom–atom interaction types.
(4) A standalone Python package pisa‐analysis to run the PISA algorithm locally on novel or modified coordinate files (Basic Protocol 3).

This approach allows researchers to perform large‐scale surveys, detailed analyses of individual PDB entries, or in silico experiments on their own models.

### Critical Parameters

The protocols described utilize specific identifiers and environmental setups to function correctly.

Basic Protocol 1. The API endpoints are keyed on three main parameters:

(1) pdbid: The 4‐character Protein Data Bank (PDB) accession code (e.g., 1iru).
(2) assemblyid: The PISA‐assigned assembly identifier. For a given pdbid, PISA may predict multiple stable assemblies (e.g., a dimer, a tetramer). This ID selects a specific one. The most stable, and thus most probable, assembly is always assigned assemblyid = 1.
(3) interfaceid: A 1‐based index for a specific pairwise interface within a given assembly.

Basic Protocol 2. Accessing data via FTP requires knowledge of the PDB ID. Data is structured in a split‐directory format based on the second and third characters of the ID (e.g., data for PDB entry 6rkw is in the /rk/ directory).

Basic Protocol 3. This protocol requires a local environment with Python 3.8+ and all package dependencies (see Basic Protocol 3, Necessary Resources). The most critical parameter is the path to the compiled PISA binary, which must be correctly set in the compile.sh script.

For analyzing assembly interfaces and interactions using pisa‐analysis, it is important to have Python 3.8+ installed, which can be obtained from the official website at: https://www.python.org/downloads/.

Additionally, ensure the installation of key Python modules: xmltodict, gemmi, xmlschema, jsonschema, lxml, and pandas. These modules are required for performing the analysis. For installation instructions for each module, please refer to its respective documentation.

### Troubleshooting

Table 1 lists issues that may arise with these protocols and their possible causes and solutions. Requests for further clarifications and error reports should be sent via email to *pdbekb_help@ebi.ac.uk*.

**Table 1:** Troubleshooting Guide for the PISA API and Pisa‐analysis Workflows

Problem	Possible cause	Solution
PISA binary error	Compilation of PISA did not work or path to the PISA binary is not set properly	Check the path to PISA binary or compile again
If PISA calculation is not running	The process might have run before, and XML files might already exist	Force PISA run with flag –force
Specific interface information missing from JSON output	Running a different version of PISA might cause this issue	Set the binary path to a version of PISA v2.1.2
API error with malformed or missing JSON response	Corrupt input CIF or rare problems related to the initial structure of the complex	This error might be due to scheduled maintenance or rarely technical issues; please try again later; if the issue persists, please email: pdbekb_help@ebi.ac.uk
Error creating JSON files after running PISA	XML files might be malformed	Check the XML files, and if needed, run again to generate corrected input files

### Understanding Results

The PISA analysis package creates JSON files that all modern programming and scripting languages can parse. Throughout the examples presented here, we demonstrated how to parse the JSON responses using Python.

Below is a summary of the key parameters found in the JSON response for a single assembly (as seen in Basic Protocol 1, step 1).

**Table:** 

Parameter	Type	Description
assembly	Object	Contains all assembly data
assembly.id	Integer	Assembly identifier

The documentation on the assembly interfaces JSON file and schema to understand the JSON responses more easily, can be found in the API specification available on Apiary at: https://pdbeapi.docs.apiary.io/#. More specifications are available from GitHub at: https://github.com/PDBe‐KB/pdbe‐pisa‐json.

Technical definitions and detailed interpretive guidelines are provided here as a reference for the parameters retrieved in Basic Protocol 1. Key parameters include:

(1) dissociation_energy (ΔGdiss): Indicates the free energy of complex dissociation (in kcal/mol). This is a primary indicator of overall assembly stability, representing the free energy difference between the associated and dissociated states. A positive value indicates that dissociation is non‐spontaneous, and an external driving force would be required to dissociate the assembly. Larger positive values denote increasing stability.
(2) Accessible surface area: This indicated the total solvent‐accessible surface area of the assembly (in Å²).
(3) Buried_surface_area (BSA): The total solvent‐accessible surface area (in Å²) buried upon the formation of all interfaces in the assembly. This quantifies the overall extent of intermolecular contact.
(4) interface_count: The total number of unique pairwise interfaces constituting the assembly.
(5) macromolecular_size: The number of macromolecular chains (e.g., protein, nucleic acid) in the assembly.
(6) Entropy: The rigid‐body entropy changes upon dissociation (in kcal/mol). The entropy change corresponds to the lowest free energy way to dissociate the complex into a set of stable complexes or monomeric units.
(7) interface_area: Calculated as the difference in total accessible surface areas of isolated and interfacing structures divided by two.
(8) Solvation energy (ΔGsolv): Indicates the solvation free energy gain upon formation of the interface, in kcal/M. The value is calculated as the difference in total solvation energies of isolated and interfacing structures. Negative ΔiG corresponds to hydrophobic interfaces, or positive protein affinity. This value does not include the effect of satisfied hydrogen bonds and salt bridges across the interface.
(9) p_value: The *p*‐value is a statistical measure of interface specificity, representing the probability of obtaining a solvation energy gain lower than observed if interface atoms were picked randomly from the protein surface. A *p*‐value = .5 means the interface is not “surprising” at all. Values > .5 indicate the interface is less hydrophobic than expected, suggesting it is likely an artifact of crystal packing. Conversely, values < .5 indicate surprising hydrophobicity, implying the interface surface is likely interaction specific. A limiting case of *p* = 0 signifies a truly unique spot on the protein surface.

### Time and Resource Considerations

The PISA API responses vary based on the input type. The API endpoints for smaller structures will return responses in 1 to 2 s. For larger structures with thousands of interfaces, such as ribosomes or viruses, the processing time may take up to 30 s. This is based on our benchmark, which was conducted on a few larger structures. The benchmark showed that the 99th percentile processing time was 16 s with a concurrency of 100.

The pisa‐analysis package performs local PISA calculations to generate assembly and interface annotations in XML format and enhanced interaction data in JSON format. Computational requirements depend on both the size and complexity of the analyzed assembly. To provide guidance on expected performance, we benchmarked the workflow using 100 representative assemblies selected from 136,614 preferred biological assemblies (Assembly ID 1) available in the PDB archive, restricted to high‐resolution experimental structures (resolution <3.5 Å) and excluding NMR entries. The benchmark was designed to evaluate performance across the full molecular‐weight distribution of these assemblies. To achieve this, preferred assemblies were grouped into four molecular‐weight ranges (10 to 100, 100 to 1000, 1000 to 10,000 and >10,000 kDa), and 25 representative structures were selected from each range.

Selection was guided not only by molecular weight but also by assembly complexity and composition. The benchmark therefore includes dimers, trimers, and larger oligomeric assemblies, as well as protein–protein, protein–nucleic acid, and nucleic acid complexes. Because the number of interfaces increases with assembly complexity, execution time depends not only on molecular weight but also on assembly composition. The table below reports assembly characteristics, including molecular weight, assembly type, composition, experimental method, and computation times required to generate the interface XML files and JSON annotation outputs. The benchmark shows that execution time is dominated by the generation of the interface XML files, whereas generation of the JSON annotation outputs is comparatively rapid across all benchmarked assembly sizes.

Benchmarking was performed using 1 CPU core per run. Production calculations within the PDBe infrastructure were performed with an initial memory allocation of 16 GB per job, which is sufficient for the majority of PDB assemblies. Larger or more complex assemblies are automatically retried with up to 60 GB of memory and a maximum runtime of 120 hr. These benchmark results provide representative estimates of the computational resources and execution times required for local analyses across the range of assembly sizes and complexities represented in the PDB archive (Table 2).

**Table 2:** Representative Benchmark of the pisa‐analysis Workflow Using 100 Biological Assemblies Spanning the Molecular‐Weight Distribution of Annotated Assemblies in the PDB Archive*^a^*

Entry ID	Assembly ID	Method class	Resolution	Oligomeric state	Assembly Type	Composition	Molecular Weight (kDa)	Range Molecular weight (kDa)	Interfaces XML generation time (s)	JSON annotation generation time (s)
8ytg	1	x‐ray	1.45	dimer	hetero	protein/protein complex	10.004	10‐100	0.29	0.069
4dzn	1	x‐ray	1.59	trimer	homo	protein structure	11.004	10‐100	0.35	0.14
3dab	1	x‐ray	1.9	dimer	hetero	protein/protein complex	12.118	10‐100	0.22	0.13
2hwn	1	x‐ray	1.6	trimer	hetero	protein/protein complex	13.337	10‐100	0.28	0.14
2pn4	1	x‐ray	2.32	dimer	hetero	RNA/RNA complex	14.683	10‐100	0.60	0.16
3ng2	1	x‐ray	1.8	dimer	homo	protein structure	16.164	10‐100	0.36	0.13
6f8f	1	x‐ray	2	dimer	hetero	protein/protein complex	17.787	10‐100	0.33	0.11
5uso	1	x‐ray	2	dimer	hetero	DNA/RNA hybrid/protein complex	19.579	10‐100	0.34	0.30
3r3k	1	x‐ray	2.2009	hexamer	homo	protein structure	21.549	10‐100	1.35	0.12
3die	1	x‐ray	1.85	dimer	homo	protein structure	23.72	10‐100	0.91	0.25
5v6i	1	x‐ray	1.7	dimer	homo	protein structure	26.107	10‐100	1.91	0.34
11ba	1	x‐ray	2.06	dimer	homo	protein structure	28.736	10‐100	0.97	0.20
1fy8	1	x‐ray	1.7	dimer	hetero	protein/protein complex	31.628	10‐100	0.85	0.17
5dv6	1	x‐ray	2.8	dimer	hetero	protein/protein complex	34.813	10‐100	0.44	0.19
4c1b	1	x‐ray	2.501	dimer	homo	protein structure	38.318	10‐100	0.60	0.31
7wqy	1	x‐ray	1.95	dimer	homo	protein structure	42.176	10‐100	0.98	0.10
5xb2	1	x‐ray	2.161	dimer	homo	protein structure	46.422	10‐100	1.53	0.25
9j47	1	x‐ray	2.38	dimer	homo	protein structure	51.095	10‐100	1.04	0.33
1lay	1	x‐ray	2.5	dimer	homo	protein structure	56.239	10‐100	0.56	0.20
9lv4	1	em	2.67	dimer	hetero	protein/protein complex	61.901	10‐100	0.62	0.10
8hpi	1	x‐ray	2.54	dimer	homo	protein structure	68.134	10‐100	5.17	0.58
6zxm	1	x‐ray	3.3	dimer	homo	protein structure	74.992	10‐100	2.59	0.43
9zr7	1	EM	3.28	trimer	hetero	protein/protein complex	82.542	10‐100	1.12	0.22
9ubd	1	x‐ray	2.53	decamer	hetero	protein/protein complex	90.853	10‐100	3.02	0.49
5vuw	1	x‐ray	2.03	dimer	homo	protein structure	99.999	10‐100	3.65	0.48
3hso	1	x‐ray	2.02	dimer	homo	protein structure	100	100‐1000	4.72	0.52
5s9v	1	x‐ray	1.9	dimer	homo	protein structure	110.068	100‐1000	6.90	0.65
4m0e	1	x‐ray	2	dimer	homo	protein structure	121.159	100‐1000	6.33	0.48
7su8	1	x‐ray	2.65	octamer	homo	protein structure	133.35	100‐1000	7.61	0.69
9kmc	1	EM	2.97	hexamer	hetero	protein/protein complex	146.776	100‐1000	5.72	0.50
5sw5	1	x‐ray	2.05	dimer	homo	protein structure	161.55	100‐1000	10.38	0.75
1egd	1	x‐ray	2.4	tetramer	homo	protein structure	177.821	100‐1000	6.27	0.88
3sym	1	x‐ray	2.4	dimer	homo	protein structure	195.719	100‐1000	2.17	0.25
9gob	1	EM	3.2	hexamer	hetero	protein/protein complex	215.402	100‐1000	6.80	0.60
7sbc	1	x‐ray	1.95	tetramer	homo	protein structure	237.118	100‐1000	13.49	1.01
8k3x	1	EM	2.86	dimer	homo	protein structure	260.993	100‐1000	1.72	0.55
9g7a	1	EM	2.12	pentamer	homo	protein structure	287.329	100‐1000	19.31	1.07
8bep	1	EM	2.29	octamer	hetero	protein/protein complex	316.195	100‐1000	8.85	0.83
8w2h	1	EM	2.6	tetramer	homo	protein structure	348.025	100‐1000	20.86	1.26
9ovu	1	EM	3.2	tetramer	hetero	protein/protein complex	383.072	100‐1000	18.38	1.38
9cas	1	EM	3.1	trimer	homo	protein structure	421.693	100‐1000	5.78	0.74
9fo6	1	EM	2.76	octamer	hetero	DNA/protein complex	464.089	100‐1000	23.92	3.50
6i9t	1	x‐ray	1.2	24‐mer	homo	protein structure	510.81	100‐1000	108.67	3.032
9dty	1	x‐ray	3.19	40‐mer	hetero	protein/protein complex	562.291	100‐1000	43.49	4.98
9jwg	1	EM	3.47	dimer	hetero	protein/protein complex	618.939	100‐1000	1.93	0.61
8tvy	1	EM	3.1	heptadecamer	hetero	DNA/RNA/protein complex	681.085	100‐1000	56.16	7.00
8jvb	1	EM	2.39	tetradecamer	homo	protein structure	749.787	100‐1000	15.22	1.95
7qvi	1	EM	3	hexadecamer	homo	protein structure	825.238	100‐1000	52.62	2.56
2tmv	1	x‐ray	2.9	98‐mer	hetero	RNA/protein complex	908.47	100‐1000	173.20	4.64
7ofq	1	EM	3.08	45‐mer	hetero	protein/protein complex	999.762	100‐1000	134.73	5.08
6zok	1	EM	2.8	22‐mer	hetero	RNA/protein complex	1000.221	1000‐10000	258.04	9.59
9hyf	1	EM	3	tetramer	hetero	protein/protein complex	1000.867	1000‐10000	49.36	3.27
9y05	1	EM	2.58	dodecamer	homo	protein structure	1100.259	1000‐10000	33.51	2.32
8tvu	1	EM	3	24‐mer	hetero	protein/protein complex	1210.492	1000‐10000	92.02	8.55
5bkn	1	x‐ray	3	117‐mer	hetero	RNA/protein complex	1216.776	1000‐10000	740.91	9.39
4nia	1	x‐ray	1.82	240‐mer	hetero	RNA/protein complex	1467.326	1000‐10000	553.85	194.64
9mm6	1	EM	2.94	octamer	homo	RNA structure	1504.648	1000‐10000	71.20	1.66
8eet	1	EM	3.1	60‐mer	homo	protein structure	1613.423	1000‐10000	468.76	9.43
9c1k	1	EM	2.68	40‐mer	hetero	protein/protein complex	1775.937	1000‐10000	369.69	17.80
9hcf	1	EM	2.85	59‐mer	hetero	RNA/protein complex	1954.314	1000‐10000	946.42	31.16
5j5b	1	x‐ray	2.8	53‐mer	hetero	RNA/protein complex	2149.293	1000‐10000	2085.63	81.35
6xzb	1	EM	2.54	54‐mer	hetero	RNA/protein complex	2164.912	1000‐10000	483.01	34.93
7msh	1	EM	3.23	55‐mer	hetero	RNA/protein complex	2366.379	1000‐10000	2327.82	45.44
7spi	1	EM	2.97	78‐mer	hetero	protein/protein complex	2478.764	1000‐10000	98.83	11.66
9t5l	1	EM	3.1	98‐mer	hetero	RNA/protein complex	2603.357	1000‐10000	318.28	7.78
6yfm	1	x‐ray	2.762	180‐mer	homo	protein structure	2865.256	1000‐10000	1216.95	7.65
8fky	1	EM	2.67	59‐mer	hetero	RNA/protein complex	4041.83	1000‐10000	754.24	72.95
8h1o	1	EM	2.67	200‐mer	hetero	protein/protein complex	5080	1000‐10000	9331.44	9.17
6sk6	1	EM	3.2	240‐mer	hetero	protein/protein complex	5592.927	1000‐10000	6514.43	38.37
7kpn	1	EM	2.5	120‐mer	hetero	protein/protein complex	6152.938	1000‐10000	3151.69	22.67
6tsu	1	EM	3.42	210‐mer	hetero	protein/protein complex	6773.107	1000‐10000	8547.71	41.71
6z8d	1	EM	2.63	120‐mer	homo	protein structure	7450.107	1000‐10000	1356.55	18.41
9fwf	1	EM	3.3	240‐mer	homo	protein structure	8201.274	1000‐10000	2197.27	10.61
8tk7	1	EM	2.53	360‐mer	hetero	protein/protein complex	9571.424	1000‐10000	3894.41	21.08
7qol	1	EM	3.33	180‐mer	hetero	protein/protein complex	9926.448	1000‐10000	4068.90	53.34
6x6s	1	EM	3.4	168‐mer	hetero	protein/protein complex	10109.835	10000+	1866.46	233.58
6hu4	1	EM	2.64	480‐mer	homo	protein structure	10133.856	10000+	2077.01	12.20
9gyi	1	EM	3.24	240‐mer	homo	protein structure	10362.103	10000+	3796.59	27.20
8vjr	1	EM	2.63	180‐mer	homo	protein structure	10427.971	10000+	4845.34	18.48
8phs	1	EM	2.82	375‐mer	hetero	protein/protein complex	10572.514	10000+	7021.00	119.23
8b12	1	EM	1.86	600‐mer	hetero	protein/protein complex	10822.218	10000+	21695.20	39.90
2gh8	1	x‐ray	3.2	180‐mer	homo	protein structure	11096.092	10000+	5005.24	31.77
6co8	1	EM	3.1	360‐mer	hetero	protein/protein complex	11405.76	10000+	20731.94	52.99
6zqu	1	EM	3.1	360‐mer	hetero	protein/protein complex	11405.915	10000+	18944.33	48.47
7kv8	1	EM	2.5	360‐mer	hetero	protein/protein complex	11609.595	10000+	24026.13	45.98
12ze	1	EM	2.6	120‐mer	homo	protein structure	11936.73	10000+	1920.79	15.62
6iat	1	EM	3.3	480‐mer	hetero	protein/protein complex	12930.577	10000+	23075.28	57.35
9rch	1	EM	2.2	120‐mer	hetero	protein/protein complex	13613.775	10000+	3863.99	46.24
7v3g	1	EM	3.3	600‐mer	hetero	protein/protein complex	14389.753	10000+	70138.31	54.68
8edu	1	EM	2.7	420‐mer	homo	protein structure	14561.443	10000+	38124.12	51.93
8b4z	1	EM	3.2	120‐mer	homo	protein structure	16330.194	10000+	5842.47	54.39
8r5y	1	EM	2.7	180‐mer	homo	protein structure	16883.164	10000+	2075.59	18.19
8ecn	1	EM	2.7	540‐mer	homo	protein structure	17837.842	10000+	59047.02	45.37
8rkc	1	EM	2.95	840‐mer	hetero	protein/protein complex	19238.98	10000+	109644.46	92.10
8zdh	1	EM	3.2	660‐mer	hetero	protein/protein complex	22748.715	10000+	163831.73	89.93
3izx	1	EM	3.1	300‐mer	hetero	protein/protein complex	31027.557	10000+	68970.28	100.22
8pkh	1	EM	3.35	1245‐mer	hetero	protein/protein complex	34644.348	10000+	291974.80	89.93
8ho3	1	EM	2.9	780‐mer	homo	protein structure	39539.293	10000+	163199.92	84.87
9gg2	1	EM	2.75	780‐mer	homo	protein structure	43465.33	10000+	> 432000.0 (timeout)	‐
9r78	1	EM	3.3	1680‐mer	hetero	protein/protein complex	102288.4	10000+	> 432000.0 (timeout)	‐

^*a*^ Abbreviations: EM: electron microscopy; X‐ray: X‐ray crystallography; homo: homomeric assembly; hetero: heteromeric assembly.

### Author Contributions

**Grisell Díaz Leines**: Formal analysis; investigation; methodology; software; validation; writing—original draft; writing—review and editing. **Paulyna Magaña**: Software; visualization; writing—original draft; writing—review and editing. **Sreenath Nair**: Resources; software; writing—review and editing. **Jennifer Fleming**: Project administration; writing—review and editing. **Mihaly Varadi**: Project administration; software; writing—original draft; writing—review and editing. **Eugene Krissinel**: Conceptualization; methodology; project administration; software; writing—review and editing. **Sameer Velankar**: Conceptualization; funding acquisition; supervision; writing—review and editing.

### Conflict of Interest

The authors declare no conflicts of interest.

## Data Availability

Documentation of the PDBe PISA API is available at: www.ebi.ac.uk/pdbe/api. The specification of the data exchange format is available at: https://pdbeapi.docs.apiary.io/#. The Jupyter notebook accompanying the protocols presented here is available as a versioned release (pisa/v1.0.0) in the pdbe‐notebooks repository at: https://github.com/PDBeurope/pdbe‐notebooks/blob/pisa/v1.0.0/pdbe_pisa_APIs/pdbe_PISA_APIs.ipynb. The macromolecular interaction data on the annotated assemblies in the PDB archive are provided via the PDBe public FTP area at: https://ftp.ebi.ac.uk/pub/databases/msd/pdb‐assemblies‐analysis/.
